# Exchange of Ahmed glaucoma valve for a Paul glaucoma implant and management of postoperative hypotony: a case report

**DOI:** 10.1097/RC9.0000000000000581

**Published:** 2026-06-25

**Authors:** Nawaf Alkuhaimi, Anas Alqurashi, Dania Bamefleh

**Affiliations:** a Glaucoma Division, King Khaled Eye Specialist Hospital and Research Center, Riyadh, Saudi Arabia; b Faculty of Medicine, Umm Alqura University, Makkah, Saudi Arabia

**Keywords:** case report, glaucoma drainage devices, hypotony, Paul glaucoma implant, uveitic glaucoma

## Abstract

**Introduction:**

Glaucoma can manifest in several forms, and some, such as uveitic glaucoma, are refractory to standard treatment. Glaucoma drainage devices (GDDs) play a crucial role in managing these diseases. The Paul glaucoma implant (PGI) is a relatively new addition to the array of available GDDs.

**Case presentation:**

We present the case of a 20-year-old woman with a history of juvenile idiopathic arthritis and uveitic glaucoma. The glaucoma was previously managed with an Ahmed Glaucoma Valve (AGV) in the left eye. Despite initial control of the intraocular pressure (IOP), it increased to 36 mmHg two years after AGV implantation, necessitating AGV removal and same-quadrant replacement with a PGI. At 2.5 months postoperatively, the patient experienced persistent IOP elevation despite maximum topical and systemic treatment for glaucoma. Ripcord removal was performed to prevent glaucomatous progression. One day later, the patient developed severe hypotony with 360° choroidal detachment within a week. The hypotony was managed with external tube ligation, resulting in resolution of choroidal detachment and improvement in visual acuity.

**Discussion:**

PGI was selected because of its small design, which may reduce the risk of hypotony. Ripcord removal should be avoided in uveitic glaucoma, as previous studies have suggested a higher incidence of hypotony. Tube ligation could be utilized for managing severe hypotony and its associated complications.

**Conclusion:**

Same-quadrant exchange with PGI may be viable after failed GDD surgery. Eyes with uveitis may have a higher risk of hypotony, especially after ripcord removal. Tube ligation effectively manages severe hypotony and maintains IOP and visual acuity.

## Introduction

Glaucoma is a widespread ophthalmic disease and one of the leading causes of visual disability worldwide[1]. Management includes various medical and surgical modalities, and new techniques are continuously being developed. Implantation of glaucoma drainage devices (GDDs) has become a common approach for achieving target intraocular pressure (IOP) in certain forms of glaucoma, particularly those refractory to treatment. The Paul glaucoma implant (PGI) is a relatively new type of GDD.

Tan *et al* reported the 2-year outcomes of 45 patients who received PGIs. They observed that 32 eyes (71.1%) achieved complete success, and only four cases (8.9%) required surgical intervention for hypotony[2]. A study by José *et al* of 24 eyes of 21 patients who received PGIs reported that there were no hypotony cases that required postoperative intervention[3].

HIGHLIGHTS

- Paul glaucoma implant can be used as a same-quadrant replacement after a failed Ahmed glaucoma valve.
- Uveitic glaucoma may carry a higher risk of hypotony with non-valved drainage devices.
- Early intraluminal ripcord removal should be avoided, particularly in uveitic glaucoma.
- Tube ligation can successfully manage severe hypotony after a Paul glaucoma implant.

Despite these studies, the full potential of PGI utilization has yet to be fully explored. We report a case in which a PGI was used to replace an Ahmed glaucoma valve (AGV) implant and describe the associated postoperative course. This case report has been presented in line with the SCARE checklist[4].

## Case presentation

We report the case of a 20-year-old woman with a history of juvenile idiopathic arthritis and uveitic glaucoma who presented to the glaucoma clinic at our tertiary hospital for follow-up. The patient was asymptomatic and had no family history of glaucoma. She had previously undergone glaucoma surgery with an AGV in the left eye 2 years earlier. She had been aphakic since childhood and had undergone anterior vitrectomy with secondary sulcus intraocular lens implantation 3 months ago. At presentation, the IOP in the left eye was 36 mmHg despite maximum medical therapy, including dorzolamide/timolol twice daily, brimonidine three times daily, bimatoprost once nightly, oral acetazolamide, oral prednisolone 10 mg daily, and mycophenolate mofetil 500 mg twice daily. The visual acuity in the left eye was 20/70.

On examination, the left eye was quiet, with a well-covered tube and plate. The anterior chamber was deep and quiet, and the intraocular lens was in place. Fundus examination showed a flat retina and advanced glaucomatous optic disc cupping. Because of the uncontrolled IOP and advanced glaucomatous changes, the patient was scheduled for AGV implant removal and simultaneous replacement with a PGI in the same quadrant, performed by a senior glaucoma consultant. Preoperatively, she was evaluated by the on-call uveitis team and received 3 days of intravenous methylprednisolone, with oral and topical steroids planned after surgery. The surgery was uneventful; the AGV and surrounding fibrous tissue were removed, and the PGI was implanted in the same quadrant with a 6-0 polypropylene suture as a ripcord.

On postoperative day 1, the patient had 20/40 visual acuity in the left eye and an IOP of 18 mmHg. The PGI tube was well positioned in the anterior chamber, and the plate was well covered. However, 2.5 months after PGI, the IOP was elevated to 35 mmHg despite continued treatment with dorzolamide/timolol twice daily, brimonidine three times daily, bimatoprost once nightly, and oral acetazolamide. Visual acuity had decreased to 20/70. Because the IOP was persistently elevated on maximum medical therapy in the setting of advanced glaucomatous optic disc cupping and a clinically quiet eye, we decided to remove the ripcord early after taking standard precautions for uveitis, including oral corticosteroids, mycophenolate mofetil 1 g twice daily, and intensive topical prednisolone acetate in the left eye.

**Figure 1.**
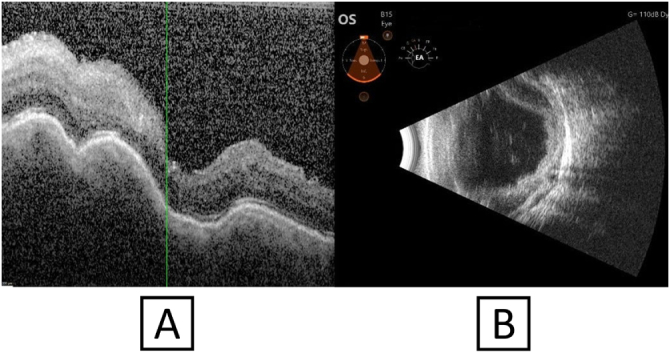
Multimodal imaging of hypotony-related posterior segment changes following ripcord removal from a Paul glaucoma implant. (A) Macular optical coherence tomography of the left eye, showing macular folds associated with hypotony. (B) B-scan ultrasonography of the left eye, showing 360° choroidal detachment.

On day 1 following ripcord removal, the visual acuity of the patient was 20/125, and the IOP was 5 mmHg. The cornea was clear, with a normal-appearing anterior chamber and no signs of inflammation. The retina showed no signs of detachment or elevation. Six days after ripcord removal, the visual acuity remained 20/125, and her IOP had decreased to 1 mmHg. Optical coherence tomography (OCT) revealed macular folds, and B-scan ultrasonography demonstrated 360° choroidal detachment with marked ocular wall thickening (Fig. 1A,B).

**Figure 2.**
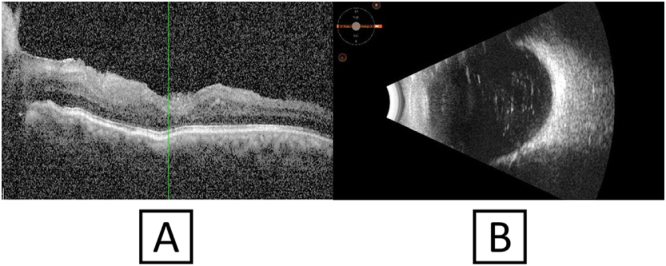
Multimodal imaging demonstrates improvement in hypotony-related posterior segment changes following the ligation of a Paul glaucoma implant tube. (A) Macular optical coherence tomography of the left eye shows improvement in macular folds secondary to hypotony following tube ligation. (B) B-scan ultrasonography of the left eye shows a flat retina and the absence of choroidal detachment.

Based on the clinical findings, we decided to perform PGI tube ligation using 7-0 Vicryl (Ethicon, Somerville, NJ, USA). The IOP and visual acuity of the patient remained unchanged on post-ligation day 1. At the 1-week follow-up, her visual acuity was 20/30, and the IOP was 12 mmHg, with a fully attached retina. Five months after ligation, her visual acuity was 20/80 and improved to 20/50 with pinhole correction. The IOP decreased to 9 mmHg after treatment with 1% topical prednisolone acetate and topical atropine, and the patient was maintained on all antiglaucoma agents. The macular folds were significantly reduced on OCT (Fig. 2A), and B-scan ultrasonography revealed an attached retina (Fig. 2B). Eighteen months after tube ligation, the patient had an IOP of 8 mmHg in the left eye and a visual acuity of 20/80 with pinhole correction. The left eye was quiet, with no signs of inflammation, and the patient reported no active complaints.

## Discussion

In this case, we describe the feasibility of using PGI as a replacement for a failed AGV in the same quadrant in uveitic eyes. The failure of the AGV was due to fibrotic tissue surrounding the capsules. Previous studies have described the exchange of AGVs for non-valved GDDs with promising results^[^5–7^]^. For example, Zuo *et al* reported outcomes of replacing failed AGVs with Baerveldt implants in the same quadrant in nine patients, three of whom were diagnosed with uveitic glaucoma[5]. Most patients achieved long-term IOP control, although two of the uveitic eyes required surgical intervention for early postoperative IOP elevation[5]. These studies support the concept of same-quadrant GDD exchange, with the advantage of preserving other quadrants for potential future surgical intervention and less manipulation, and therefore less inflammatory reaction, in uveitic eyes.

We selected the PGI over the AGV or Baerveldt implant, in part because of the newer device design. Compared with the AGV and Baerveldt implants, the PGI has a smaller internal lumen (0.127 mm vs. 0.305 and 0.320 mm, respectively) and a smaller external tube diameter in the anterior chamber (0.467 mm vs. 0.635 and 0.640 mm, respectively), which may reduce the risk of early postoperative hypotony[8]. This may be particularly relevant in uveitic eyes, in which glaucoma surgery carries a higher risk of hypotony and ciliary body shutdown[9]. Ramdas *et al* reported a higher transient hypotony in uveitic eyes that underwent GDD implantation compared to non-uveitic eyes (15.8% vs. 8.2%), although this difference did not reach statistical significance[10]. Similarly, Sinha *et al* reported that hypotony was the most common cause of failure in uveitic eyes with the Baerveldt implant[11]. However, the study did not specify whether it occurred immediately after implantation or after intraluminal ripcord removal.

The use of PGI in uveitic glaucoma remains limited, and no head-to-head studies have compared PGI with other GDDs for uveitic glaucoma. Recent studies have found favorable outcomes and reported low rates of failure and persistent hypotony[12]. Despite these encouraging results, our patient developed severe clinical hypotony associated with 360° choroidal detachment, particularly following ripcord removal 2.5 months after PGI implantation.

The optimal timing for ripcord removal in non-valved GDDs varies among studies[13]. Recent PGI literature recommends avoiding ripcord removal before 8–12 weeks and suggests that delaying removal for approximately 4–5 months may be preferable^[^10,13^]^. Uveitic eyes are more prone to hypotony-related complications and inflammatory reactions, and these considerations may be even more important. Richardson *et al* recommended maintaining uveitic eyes on glaucoma medication and avoiding early ripcord removal, as abrupt increases in aqueous flow can occur[12]. Hence, it is possible that a longer duration of stent retention may have mitigated the risk of hypotony in our case.

When clinically significant hypotony develops after ripcord removal in non-valved GDDs, tube ligation with non-absorbable sutures is a logical rescue approach because it restricts aqueous outflow and thereby increases IOP. Previous studies have reported a high success rate of Baerveldt implants after ligating the tube, while data on PGI remain limited^[^14,15^]^. Studsgaard *et al* reported two cases of clinical hypotony after Ripcord removal in PGI, both treated with tube ligation and successfully managed hypotony[16]. These findings are consistent with our case, in which tube ligation elevated the IOP to the normal range and managed the 360° choroidal detachment, preserving visual acuity.

This case report discusses the feasibility of using PGI to exchange a failed GDD in the same quadrant. Hypotony, especially in uveitic eyes, should be considered, and ripcord removal should be avoided in the early phases. Tube ligation to manage hypotony maintained controlled IOP and visual acuity after severe choroidal detachment. However, this report reflects a single complex uveitic eye; therefore, larger studies are needed to better define PGI use and the optimal timing of ripcord removal in uveitic glaucoma.

## Conclusion

Uveitis may be a risk factor for hypotony after ripcord removal from the PGI. We report a case of AGV failure secondary to endplate fibrosis, followed by same-quadrant replacement with a PGI and the subsequent postoperative course. We also describe the postoperative IOP increase and severe hypotony associated with early ripcord removal at 2.5 months, as well as our use of tube ligation for hypotony management. To the best of our knowledge, this is the first report of a valved GDD being replaced with a PGI in a case of uveitis. This case report adds to the limited literature on the use of PGI in uveitic glaucoma and may support future studies investigating the optimal time for ripcord removal and the management of the hypertensive phase in uveitic glaucoma.

## Data Availability

All data relevant to this case report are included in the article.

## References

[1] Quigley HA. The number of people with glaucoma worldwide in 2010 and 2020. Br J Ophthalmol 2006;90:262–67. 16488940 10.1136/bjo.2005.081224 PMC1856963

[2] Tan MCJ Choy HYC Koh Teck Chang V. Two-year outcomes of the Paul glaucoma implant for treatment of glaucoma. J Glaucoma 2022;31:449–55. 35180153 10.1097/IJG.0000000000001998 PMC9148669

[3] José P Barão RC Teixeira FJ. One-year efficacy and safety of the PAUL glaucoma implant using a standardized surgical protocol. J Glaucoma 2022;31:201–05. 34930872 10.1097/IJG.0000000000001969

[4] Kerwan A Al-jabir A Mathew G. Revised Surgical CAse REport (SCARE) guideline: an update for the age of Artificial Intelligence. Prem J Sci 2025;10:100079.

[5] Zuo W Lesk MR. Surgical outcome of replacing a failed ahmed glaucoma valve by a Baerveldt glaucoma implant in the same quadrant in refractory glaucoma. J Glaucoma 2018;27:421–28. 29462014 10.1097/IJG.0000000000000912

[6] Jacobson A Bohnsack BL. Ahmed to Baerveldt glaucoma drainage device exchange in pediatric patients. BMC Ophthalmol 2023;23:310. 37434139 10.1186/s12886-023-03074-1 PMC10334620

[7] Khan AM Abdalla Elsayed MEA Malik R. Case report: inferior valved for non-valved glaucoma drainage device exchange for glaucoma control and cosmesis. Front Ophthalmol (Lausanne) 2024;4:1361898. 38984121 10.3389/fopht.2024.1361898 PMC11182133

[8] Qin Q Zhang C Yu N. Development and material characteristics of glaucoma surgical implants. Adv Ophthalmol Pract Res 2023;3:171–79. 38106549 10.1016/j.aopr.2023.09.001 PMC10724012

[9] Wang Q Thau A Levin AV. Ocular hypotony: a comprehensive review. Surv Ophthalmol 2019;64:619–38. 31029581 10.1016/j.survophthal.2019.04.006

[10] Mendel L Tan JCK Aguilar Munoa S. Outcomes of intraluminal ripcord removal from Paul glaucoma implants. Br J Ophthalmol 2025;109:1246–51. 40588330 10.1136/bjo-2024-326947 PMC12573402

[11] Sinha S Ganjei AY McWatters Z. Ahmed versus baerveldt glaucoma drainage device in uveitic glaucoma: a retrospective comparative study. J Glaucoma 2020;29:750–55. 32590449 10.1097/IJG.0000000000001583

[12] Richardson J Tacea F Yu J. The PAUL Glaucoma Implant in the management of uveitic glaucoma-3-year follow-up. Eye (Lond) 2025;39:931–37. 39623106 10.1038/s41433-024-03527-x PMC11933445

[13] Millá E Moreno-montañés J Duch S. Predictive factors for the success of the Paul glaucoma implant: a one-year multicenter prospective study. Sci Rep 2025;15:38284. 41184303 10.1038/s41598-025-18369-0 PMC12583770

[14] Stein JD McCoy AN Asrani S. Surgical management of hypotony owing to overfiltration in eyes receiving glaucoma drainage devices. J Glaucoma 2009;18:638–41. 19826394 10.1097/IJG.0b013e31819aa4e0

[15] Mavrommatis MA Dangda S Sidoti PA. Downsizing a Baerveldt glaucoma implant for the management of persistent postoperative hypotony: a case series. J Glaucoma 2019;28:1019–22. 31517761 10.1097/IJG.0000000000001365

[16] Studsgaard A Nielsen SE Telinius N. One tube for all: 1-year outcomes after transition to Paul glaucoma implant at a tertiary centre. Acta Ophthalmol 2025;103:461–68. 39853904 10.1111/aos.17443 PMC12069969

